# Effectiveness and safety of massage for knee osteoarthritis: A protocol for systematic review and meta-analysis

**DOI:** 10.1097/MD.0000000000022853

**Published:** 2020-10-30

**Authors:** Siyu Qin, Zhenhai Chi, Yuanyi Xiao, Daocheng Zhu, Genping Zhong, Wei Xu, Xilin Ouyang, Jun Li, Pan Cheng, Ting Yu, Haiyan Li, Lin Jiao

**Affiliations:** a College of Acupuncture-Moxibustion and Tuina, Jiangxi University of Traditional Chinese Medicine; b The Affiliated Hospital of Jiangxi University of Traditional Chinese Medicine, Nanchang, China.

**Keywords:** knee osteoarthritis, massage, protocol, systematic review

## Abstract

**Background:**

Knee osteoarthritis (KOA), a concerning public health problem, seriously threatens well being of human beings. At present, studies have shown that massage therapy is effective in relieving related symptoms of KOA. However, the evidence of massage for KOA has not been systematically evaluated. Therefore, the study is conducted to systematically assess the reliability of patients with KOA treated by massage.

**Methods:**

We will retrieve the relevant literature of massage for KOA from PubMed, Cochrane Library, EMBASE, Web of Science, Wanfang, Chongqing VIP, CNKI, and Chinese Biomedical Literature Database from the establishment of the databases to August 1, 2020. Two researchers will independently perform the screening of literature and extract the basic information of the data. In addition, RevMan V.5.3 software will be used for data analysis.

**Results:**

The study will comprehensively assess the effect of massage for KOA.

**Conclusion:**

The study will provide comprehensive evidence for evaluating whether massage therapy is useful in treating patients with KOA.

**INPLASY registration number:**

INPLASY202080115.

## 1 Introduction

Osteoarthritis is the most prevalent joint disorder in the world, and the site most often affected by osteoarthritis is the knee.^[1,2]^ Knee osteoarthritis (KOA) is a degenerative disease that is characterized by pain, swelling, stiffness, and motor dysfunction.^[3]^ Currently, the incidence of KOA in the elderly is quite high, and it is reported that 30% to 50% of the elderly population more than 60 years of age suffer from KOA.^[4]^ With the coming of the aging of the world population, KOA has become a concerning public health problem that not only brings an increasing social and economic burden but also threatens the physical and mental health of patients as well as reduces the quality of life of the elderly.^[5,6]^ Meanwhile, studies have shown that besides joint discomfort, patients with knee osteoarthritis are more likely to have poor sleep and feel anxiety and depression compared with healthy people.^[6–9]^ Therefore, it is very important to find effective treatments for improving patients’ quality of life and reducing the medical burden.

At present, the common treatments for KOA mainly include health education, pharmacological and non-pharmacological treatments as well as surgery.^[10,11]^ Drug treatment, as a method recommended by clinical guidelines, is useful in alleviating pain and inflammation of KOA.^[12,13]^ However, long-term use of drugs may bring a variety of adverse impacts such as hepatic toxicity, renal toxicity, and gastrointestinal complications.^[14]^ Besides, although knee replacement is effective for advanced KOA, some patients are not able to undergo the operation due to economic burden and physical condition.^[15]^ Hence, seeking a safe and effective alternative treatment is urgently required to alleviate the distress of patients who have been diagnosed with KOA.

Massage, as 1 of the most widely used Complementary and Alternative Medicine therapies, is defined a method of touching or manipulating body soft tissues by hand to provide comfort.^[16]^ Compared to pharmacological therapy and surgery, massage has unique advantages because of its characteristics of high safety, low-cost, and easy access.^[17]^ It is reported that 15.4 million Americans used massage therapy to treat KOA in 2012.^[18]^ Besides, a large number of trials have shown that massage therapy is an effective non-drug intervention that can improve pain, stiffness, and functional status of patients diagnosed with KOA.^[17,19–21]^ Previous studies have also demonstrated that massage can relieve the related symptoms of knee osteoarthritis by promoting the blood circulation around the joint, improving the tension of the muscle as well as increasing the flexibility of the joint.^[22,23]^

To our knowledge, there is no systematic review discussing whether massage therapy is safe and effective for patients who have been diagnosed with KOA. Therefore, we perform this protocol to comprehensively assess the effect of massage for KOA.

## 2 Methods

### 2.1 Study registration

This protocol was registered on the International Platform of Registered Systematic Review and Meta-Analysis Protocols (INPLASY) on August 27, 2020 (registration number INPLASY202080115). We will strictly perform this protocol by following the Preferred Reporting Items for Systematic Reviews and Meta-Analyses Protocol (PRISMA-P)^[24]^ statement guidelines.

### 2.2 Inclusion criteria for study selection

#### 2.2.1 Type of studies

We will only include randomized clinical trials (RCTs) about massage for KOA, with language restrictions in English or Chinese. Case report, experience report, and laboratory studies will not be included.

#### 2.2.2 Types of Participants

All patients with KOA will be included without limitation of age, race, sex, economic level, and severity.

#### 2.2.3 Types of interventions

##### 2.2.3.1 Experimental interventions

The intervention of the experimental group will only include massage therapies, which mainly include general massage, acupressure, Chinese massage, relaxation, manual lymphatic drainage, and so on. There is no limitation on the methods, duration, and frequency of massage.

##### 2.2.3.2 Control interventions

The interventions of the control group will involve any therapies other than massage (eg, drug therapy, placebo, acupuncture, routine care, etc)

#### 2.2.4 Types of outcome measures

##### 2.2.4.1 Primary outcomes

The Western Ontario and McMaster Universities Osteoarthritis Index scale.

##### 2.2.4.2 Additional outcomes

(1) Visual analog scale.
(2) Symptom score.
(3) Lysholm knee scoring scale.
(4) Adverse events.

### 2.3 Search methods

RCTs of massage for KOA will be searched from PubMed, Cochrane Central Register of Controlled Trials, EMBASE, Web of Science, Chinese Biomedical Literature Database, Wanfang Database, Chongqing VIP Database, and Chinese National Knowledge Infrastructure from inception to August 1, 2020. In addition, The Clinicaltrials.gov, Chinese Clinical Trial Registry will also be carefully retrieved to obtain unpublished or ongoing relevant studies. The specific PubMed retrieval strategy is presented in Table 1.

**Table 1:**
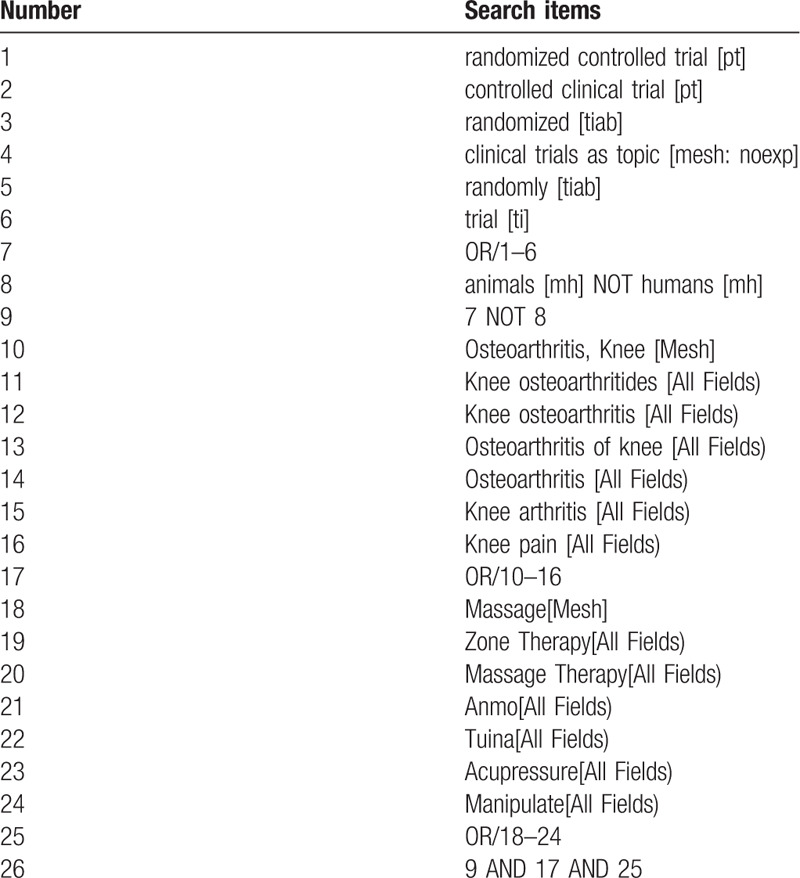
Search strategy used in PubMed database.

### 2.4 Data collection and analysis

#### 2.4.1 Selection of studies

**Figure 1:**
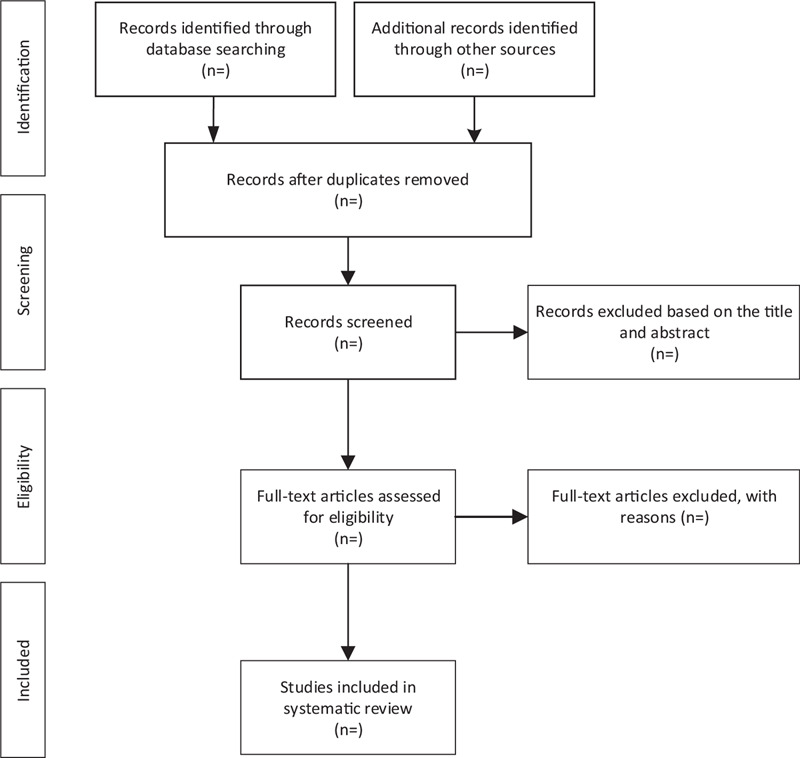
Flow diagram of study selection process.

We will import all retrieved literature into EndNote software (V.x9) and remove duplicate literature. The 2 qualified reviewers (SQ and ZC) will make a preliminary screening to exclude irrelevant literature by reading the title and abstract independently. Then, the researchers (SQ and ZC) will eventually decide whether the literature will be included in the study by reading the full text. Finally, any divergence arising from the above process will be solved or discussed through the third researcher (LJ). The specific literature screening flow chart is shown in Figure 1.

#### 2.4.2 Data extraction and management

The researchers (SQ and YX) will extract relevant data from the included literature, mainly including the following information:

(1) Research Characteristics: Publication year, study title, basic information of the first author.
(2) Patient information: Sex, age, the severity of KOA, duration of disease, nationality, sample size.
(3) Study methods: Randomization, allocation concealment, blinding, result analysis method.
(4) Intervention: The method of massage, treatment sites, and frequency.
(5) Outcomes measurement: Included primary and additional outcomes.

### 2.5 Evaluation of bias risk in included studies

The 2 independent researchers (SQ and ZC) will use the Cochrane bias risk assessment tool^[25]^ to evaluate the risk of bias of the included RCTs. It includes 7 domains: random sequence generation, allocation concealment, blinding of participants and personnel, blinding of outcome assessment, incomplete outcome data, selective reporting, and other bias. In addition, the risk of bias can be categorized as high, low, and unclear risk bias levels. If there is any divergence, it will be resolved by consulting a third researcher (LJ).

### 2.6 Data synthesis

RevMan 5.3 software will be used to perform the statistical analysis. For discontinuous variables, the risk ratio (RR) with 95% confidence interval (CI) will be selected. For continuous variables, the Weighted Mean Difference (WMD) with 95% CI will be selected when the measurement instruments are the same, and the Standardized Mean Difference (SMD) with 95% CI will be selected when the measurement instruments are different. We will use the fixed-effect model if there is no obvious heterogeneity (*P* > .1 or *I*^2^ < 50%). We will use the random-effect model if there is an obvious heterogeneity (*P* ≤ .1 or *I*^2^ ≥ 50%), and subgroup analysis or sensitivity analysis will be carried out to find the possible causes of the heterogeneity between groups.

### 2.7 Management of missing data

It is necessary to contact the relevant author to get the completed information when there are incomplete or missing data in the included trials. If the missing data is still not available, we will abandon the missing data and analyze the existing data.

### 2.8 Subgroup analysis

If necessary, we will conduct the subgroup analysis to reduce the clinical heterogeneity between groups in terms of differences in the types of massage, gender, treatment duration, and frequency, and so on.

### 2.9 Sensitivity analysis

To monitor the reliability of the meta-analysis results, we will conduct a sensitivity analysis to exclude low-quality trials if the included trials are sufficient.

### 2.10 Assessment of reporting biases

We will adopt funnel plots to assess publication bias when the included RCTs exceed 10. Besides, we will use the Egger test to explore the potential causes of publication bias when the funnel plots are asymmetric.

### 2.11 Quality of evidence

Two researchers (SQ and DZ) will use the Grading of Recommendations Assessment, Development and Evaluation (GRADE)^[26]^ to independently evaluate the quality of evidence which will be graded into high, moderate, low, and very low levels.

### 2.12 Ethics and dissemination

The patients’ privacy is not involved in the study, so ethical approval is not needed. In addition, the systematic review will be disseminated in a peer-reviewed journal.

## 3 Discussion

Knee osteoarthritis, 1 of the main causes of disability worldwide, seriously threatens the physical and mental health of people.^[27]^ Even though pharmacological treatments and surgery are effective in alleviating the symptoms of patients who have been diagnosed with KOA, they may have some side effects. Massage as a complementary and alternative therapy is widely used to relieve the pain of patients who are diagnosed with KOA due to its features of high safety, low-cost, and easy access.^[17]^ Previous studies have also confirmed that massage therapy is useful in improving pain, stiffness, and functional status for patients with KOA.^[17,19–21]^ However, at present, the evidence of massage for KOA lacks comprehensive system evaluation. So, we hope that the study can provide valuable information to patients, physicians, and health authorities.

However, the study may have some potential limitations. First, greater heterogeneity may exist due to different massage methods. Second, the quality of the study may be affected because we only include clinical trials published in Chinese or English.

## Author contributions

**Data collection:** Siyu Qin, Zhenhai Chi.

**Funding acquisition:** Lin Jiao.

**Methodology:** Siyu Qin, Yuanyi Xiao.

**Software:** Daocheng Zhu.

**Supervision:** Lin Jiao.

**Writing – original draft:** Siyu Qin, Zhenhai Chi.

**Writing – review & editing:** Lin Jiao.
